# Improving Resident Doctors' Preparedness for General Medical On-Call Shifts Through Structured Induction Resources: A Quality Improvement Project at a Large UK Teaching Hospital

**DOI:** 10.7759/cureus.109166

**Published:** 2026-05-19

**Authors:** Charlotte Brighton, Benjamin Anderson

**Affiliations:** 1 General Medicine, North Manchester General Hospital, Manchester, GBR; 2 Palliative Care, Salford Royal NHS Foundation Trust, Salford, GBR

**Keywords:** doctor preparedness, general medical on-calls, induction, induction materials, pdsa cycles

## Abstract

Resident doctors frequently report feeling underprepared when commencing general medical on-call duties. This is important as poor preparedness contributes to anxiety, inefficiency, and potential risks to patient safety. It has been found that preparedness is strongly influenced by the quality of local induction.

This quality improvement project aimed to improve resident doctors' self-reported preparedness for their first general medical on-call shift at Salford Royal Hospital through the introduction of structured induction resources. Two Plan-Do-Study-Act (PDSA) cycles were undertaken between October 2024 and March 2025. Preparedness was assessed using questionnaires distributed to FY1 to ST7 grades before and after interventions. The first comprised an optional in-person hospital tour, and the second involved the development and dissemination of a structured induction booklet addressing on-call roles, escalation pathways, handover arrangements, and hospital logistics.

At baseline, four of 31 doctors (12.9%) reported feeling prepared for their first on‑call shift. PDSA cycle 1 had minimal impact due to low uptake and was not felt relevant to the group of doctors rotating. However, following the introduction of the induction booklet in PDSA cycle 2, 18 of 30 doctors (60%) reported feeling prepared (p<0.001). Improvements were also seen in the understanding of on‑call roles, the acute medical take, and escalation processes. However, hospital orientation remained suboptimal.

Structured induction resources improve resident doctors' perceived preparedness for general medical on-call duties. While written induction materials are low-cost and scalable, our findings suggest that a variety of structured induction materials are required to ensure adequate preparation. Furthermore, ensuring induction materials within a timely manner is crucial and an area for further improvement.

This project did not assess clinical outcomes, but it is of interest whether improving preparedness improves clinical performance.

## Introduction

Preparedness for clinical practice is a fundamental component of safe, effective, and sustainable healthcare delivery. Poor preparedness among doctors has been associated with increased anxiety, cognitive burden, and stress, which may impair clinical decision‑making and efficiency, particularly in high‑pressure environments such as acute medical on‑call work [1-3]. The "Black Wednesday" phenomenon, describing increased in‑hospital mortality following the annual August changeover of doctors, highlights the potential patient safety implications of inadequate preparation for new clinical roles [1].

Unpreparedness is not limited to newly qualified doctors. Trainees at all grades report difficulties when transitioning into new hospitals, roles, or rotas, particularly when local systems, expectations, and escalation pathways are unfamiliar [3,4]. Induction is therefore a critical mechanism for supporting preparedness during these transitions. Qualitative work by Burridge et al. demonstrated that residents' preparedness for acute care develops through a combination of formal induction, informal support, and experiential learning, with inconsistent induction contributing to anxiety and delayed confidence development [5]. Where induction is poorly structured or absent, doctors rely on ad hoc learning during on‑call shifts, increasing stress and potential risk to patient safety [6].

National data mirror these concerns. Research commissioned by the General Medical Council (GMC) demonstrates wide variation in the content, timing, and effectiveness of local induction, with many doctors reporting that induction does not adequately prepare them for out‑of‑hours or acute care [6,7]. Conversely, structured, role‑specific induction programmes have been shown to improve doctors' confidence, understanding of local systems, and perceptions of safety [8].

At our hospital, informal feedback from resident doctors (FY1 to ST7) highlighted widespread dissatisfaction with induction for general medical on‑call shifts with many reporting they felt unprepared for their first on‑call shift. Key concerns included a lack of clarity regarding role expectations, escalation pathways, handover arrangements, and hospital geography. Unpreparedness in this context represents a risk to doctor wellbeing, morale, and patient safety. Salford Royal Hospital is a large tertiary teaching hospital in the North West of England with a wide geographic footprint encompassing multiple buildings and numerous specialist services. Resident doctors from a range of specialties provide general medical out‑of‑hours cover to medical wards and to non‑medical wards hosting medical outliers, in addition to responding to medical emergencies and cardiac arrests overnight.

In response, a quality improvement project was initiated using the Plan-Do-Study-Act (PDSA) methodology [9]. The primary objective was to improve resident doctors' self‑reported preparedness for their first general medical on‑call shift through the implementation of structured induction interventions (a hospital orientation tour and a written induction handbook). The primary outcome measure was the proportion of doctors reporting preparedness (defined as a Likert score ≥4). The project aimed to increase this proportion from a baseline of 12.9% to at least 50% following intervention.

## Materials and methods

Setting and study design

This single‑centre quality improvement project was conducted at Salford Royal Hospital in Salford, UK, between August 2024 and March 2025. Following an initial baseline data collection, the project employed a pre‑ and post‑intervention design using two sequential PDSA cycles [9]. Baseline data collection was taken from a cohort of doctors who were working on the general medical on-call rota between August and November 2024 (31 doctors responded; however, one respondent did not provide information on the secondary outcome measure or self-reported orientation). PDSA cycle 1 evaluated the response of resident doctors rotating into medical posts in December 2024 (25 rotating doctors were identified, but only one doctor attended the tour and responded). PDSA cycle 2 evaluated a new intake of doctors commencing posts in February 2025 (30 responding doctors).

The project team comprised resident doctors with general medical on-call experience, ranging from FY1 to ST5 levels. There was a mixture of locally employed and doctors in training. This was felt vital in order to capture the concerns of all rotating doctors at various stages of their careers and design interventions accordingly. All of the doctors in our team worked on general medicine on calls. We therefore had experience of the shifts we were trying to improve preparedness for, with the result that interventions were relevant and focused.

Involvement with key stakeholders such as the postgraduate medical education team and acute medical consultants was vital. As induction is led by these two groups, any intervention suggested had to be carried out with their knowledge and approval to guarantee success. Our meetings involved a member of the postgraduate meetings to ensure interventions were feasible and provide an ally for implementation.

This approach also ensured sustainability. Resident doctors on rotational contracts and not certain to work in the general medical department moving forward comprised the main quality improvement team, and this approach ensured that permanent staff in charge of induction both saw the benefits of any change and felt ownership to continue this in the future.

Additionally, involvement of trust bodies such as the postgraduate medical education meant content could be provided at scale to all resident doctors starting at the trust. This avoided the reliance on a small pool of doctors' contacts, and accessibility and equity for residents starting at the hospital could be guaranteed through trust channels and resources. 

Measurement and data collection

Self-reported preparedness was selected as the primary outcome measure, as it is frequently used in evaluations of induction programmes and has been linked to doctor wellbeing and perceived safety [5,10]. Preparedness was measured using a locally developed, non‑validated questionnaire incorporating Likert‑scale items (5‑point scale from strongly disagree to strongly agree) and free‑text comments. The questionnaire assessed self‑reported preparedness for the first general medical on‑call shift, alongside understanding of on‑call shift types, the acute medical take, handover processes, escalation pathways, and hospital orientation. The full questionnaires used can be seen in Appendix A and Appendix B and were carefully designed by clinicians experienced in local general medical on‑call work to ensure face validity and relevance to real‑world practice.

Online questionnaires were distributed electronically via trust email and were also advertised via posters with QR codes displayed in key on‑call areas. Questionnaires were also printed and distributed at medical handovers. Questionnaires following the interventions were collected one month following the changeover dates. Responses remained open for the online questionnaires for two weeks. No validated scale specific to UK general medical on‑call preparedness exists, and formal permission for use of external proprietary instruments was not required.

Interventions

Following initial data collection to provide important resident doctor feedback, two interventions were designed.

PDSA Cycle 1

An optional in‑person hospital orientation tour was offered to 25 resident doctors starting general medicine at the December 2024 changeover. The intervention aimed to improve geographical orientation, identified at baseline as a frequent source of difficulty. The tour was advertised via email (sent by the quality improvement project team) one week prior to the date and again the day prior.

PDSA Cycle 2

A structured written induction booklet ("A Survival Guide to: Medical On‑Call Shifts at Salford Royal Hospital") was developed by resident doctors and disseminated digitally one week prior to the February 2025 changeover via trust email, within group chats via messaging platforms, and via QR codes on posters that were placed in clinical areas and handover rooms. The hypothesis was that a written resource would be more accessible, scalable, and usable across time.

The handbook included information for all grades on the location and expectations of handovers, the different types of on-call shifts and the expectations for these, the bleeps carried by the on-call team, and key information about the cardiac arrest team. It also included information on the wards covered by the on-call team and how the "Hospital at Night Team" operates and supports the on-call team and allocates workload via an online system. The booklet provided an overview of how the acute medical take operates locally and included a guide to local referral processes. Key information regarding carparking, rest and food facilities, and how to report absences were also included.

Statistical analysis

Questionnaires sent out consisted of both quantitative and qualitative questions with a mix of Likert scales, multiple-choice answers, and open-ended questions being used, seen in Appendix A and Appendix B. Descriptive statistics were used to summarise questionnaire responses.

For inferential analysis, categorical outcomes were compared between baseline and cycle 2 using Fisher's exact test due to the small sample size. Likert‑scale items were dichotomised into agree (scores of 4-5) versus neutral or disagree (scores of 1-3) for analysis. This approach was chosen to simplify interpretation and allow comparison of meaningful agreement in self-reported preparedness and secondary measures. Statistical significance was defined as p<0.05. Inferential analysis was not performed for PDSA cycle 1 due to the insufficient uptake of the intervention.

For outcomes relating to hospital orientation, responses were similarly dichotomised into "frequent disorientation" (every shift/every other shift) versus "infrequent disorientation" (rarely/never), enabling the consistent application of Fisher's exact testing. Given that only 30/31 respondents in the baseline data gave an answer regarding orientation, the cohort size was altered to allow consistency and as alternative statistical tests were not felt to be appropriate due to the small sample size.

## Results

**Figure 1:**
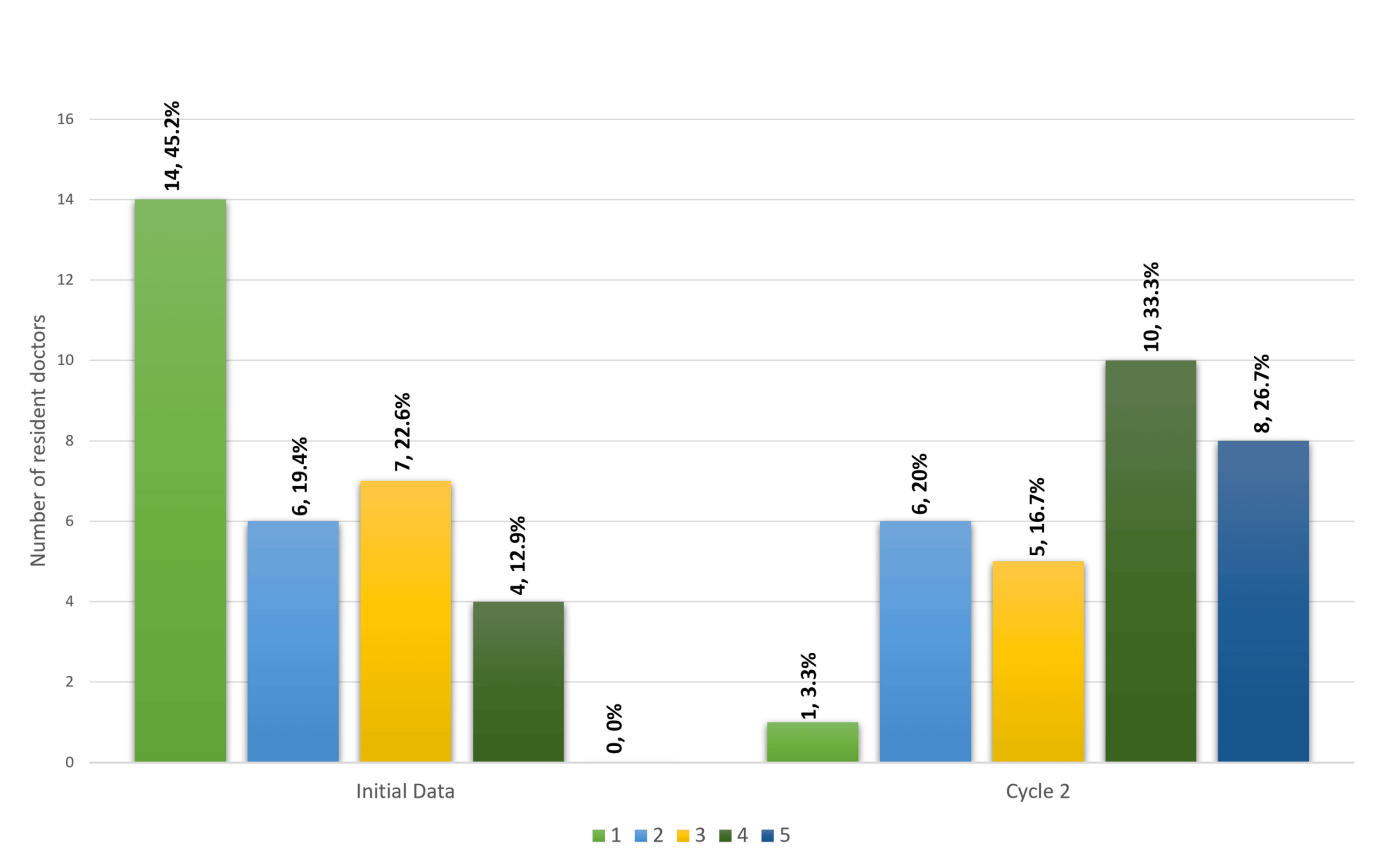
Bar chart showing resident doctors' responses to how much they agreed (using Likert scale) that they felt prepared to start on-call shifts in the initial data collection and in cycle 2

The proportion of residents reporting preparedness for their first general medical on‑call shift increased from 4/31 (12.9%) at baseline to 18/30 (60%) following the dissemination of the induction booklet in cycle 2. This difference was statistically significant (p<0.001), representing an absolute increase in preparedness of 47.1%. The level respondents who agreed or disagreed with the statement "I felt prepared to safely start on‑call duties" in the baseline data and cycle 2 can be seen in Figure 1.

Between the baseline data collection and cycle 2, secondary measures also showed improvement, and the data are seen in Table 1. Agreement with the statement "I understood the different on‑call shifts at Salford Royal and the role I needed to do" increased from 6/31 respondents (19.4%) at baseline to 15/30 respondents (50%) in cycle 2 (p=0.012). Agreement with "I understood how the acute medical take worked at Salford Royal" increased from 3/31 (9.7%) to 15/30 (50%) (p<0.001). Furthermore, agreement with "I knew how to access senior support out of hours" increased from 9/31 (29%) to 21/30 (70%) (p=0.002).

**Table 1:** Number of resident doctors who agreed with the following statements, including the primary outcome of feeling prepared and also secondary outcomes

Outcome assessed	Baseline (n=31)	Cycle 2 (n=30)	P-value
	Number of doctors who agreed, N (%)	Number of doctors who agreed, N (%)	
I understood the different on-call shifts at Salford Royal and the role I needed to do	6 (19.4%)	15 (50%)	0.016
I understood how the acute medical take worked at Salford Royal	3 (9.7%)	15 (50%)	<0.001
I knew how to contact my team including seniors	9 (29%)	21 (70%)	0.002
I felt prepared to safely start on‑call duties	4 (12.9%)	18 (60%)	<0.001

In the baseline data collection, despite 31 responses in total for the primary outcome and the above secondary outcomes, only 30 resident doctors provided a response to their perceived orientation. Although incomplete, the data were included, and the change in cohort size has been accounted for statistically. No statistically significant improvement was observed in hospital orientation, with similar proportions frequently getting lost or asking for directions in the first month, as seen in Table 2. 

**Table 2:** Number of resident doctors who frequently got lost or had to ask for directions within the first one month in baseline data and cycle 2

Question	Baseline (n=30)	Cycle 2 (n=30)	P-value
	Number of doctors, N (%)	Number of doctors, N (%)	
Frequent disorientation (Those who reported getting lost every shift/every other shift or needing to ask for directions)	22 (73.3%)	23 (76.7%)	1.00

**Figure 2:**
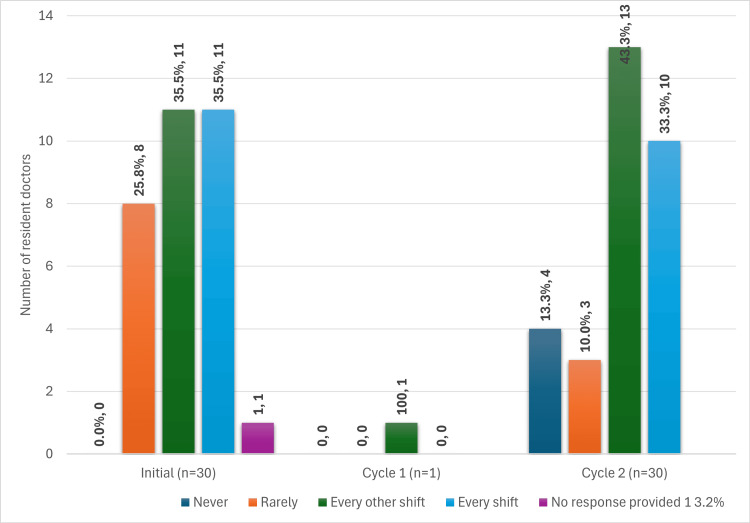
Orientation as a proxy of resident doctors' preparedness: bar chart showing resident doctors' responses to how often, in their first month, did they get lost or had to ask for directions while on call (baseline data, cycle 1, and cycle 2)

In cycle 2, 23/30 resident doctors (76.7%) report getting lost or needing to ask for directions at least every other shift during their first month, compared with 22/30 complete respondents (73.3%) at baseline (p=1.00). The full data from baseline data, cycle 1, and cycle 2 are seen in Figure 2.

In PDSA cycle 1, only 1/25 residents attended the orientation tour. The one attending resident reported getting lost every other shift, as seen in Figure 2. Owing to the extremely low uptake of the hospital orientation tour during PDSA cycle 1 (1/25 eligible doctors, 4%), inferential statistical analysis was not undertaken for this intervention.

Qualitative data

At baseline, respondents frequently described an absence of formal medical on‑call induction, leading to confusion and anxiety. One FY1 doctor described feeling unprepared despite clinical competence, noting that "I felt ready to deal with the jobs themselves, but was often confused by which handover was where and how to find things". Several respondents highlighted the emotional impact of entering on‑call roles without structured induction, with one senior trainee stating that "Radio silence about on‑calls made me feel very anxious and unsupported even as a senior trainee".

In cycle 1, the single respondent reported that the tour was "Useful in helping prepare" but suggested that "On-calls should be explained in addition to the tour".

In cycle 2, post‑intervention feedback highlighted the perceived value of the structured induction booklet with respondents describing it as "Very easy to understand and very useful" and "The most useful part of induction". However, responses identified barriers that limited the booklet's impact. Several respondents reported receiving the booklet late or being unaware of its availability, with comments such as "Received the booklet after I started". These align with quantitative findings showing residual unpreparedness despite improved materials.

## Discussion

Our project aimed to improve preparedness for resident doctors. We felt that increasing preparedness through induction would reduce resident doctor anxiety and stress and improve safety. The assumption that transitions into new clinical environments are associated with increased stress and reduced confidence among junior doctors, with potential implications for patient safety, is well reported [5,11]. While it is widely suggested that a more prepared doctor is a safer doctor, there remains limited evidence demonstrating a direct relationship between preparedness and objective clinical effectiveness [5,12].

Doctors across the whole division of medicine were included within this project, and outcome measures would span multiple disease groups, being influenced by many non‑clinical factors. Measuring the impact of induction on clinical outcomes was therefore beyond the scope of this project. This reflects recognised methodological limitations seen in previous induction studies [5]. Therefore, further research is recommended to determine whether improved induction for rotating doctors leads to measurable clinical outcomes.

The second PDSA cycle demonstrated a 47.1% increase in perceived preparedness, supporting evidence that induction resources written by resident doctors are more likely to address practical, shift‑relevant knowledge gaps [13,14]. Dissemination through postgraduate medical education ensured appropriate governance and equitable access, in keeping with GMC guidance [15]. The handbook was available via a PDF file which was shared electronically via email as well as via QR codes included on posters in key on-call areas. It could be shared easily via digital messaging. Digital dissemination improved usability during on‑call shifts, aligning with studies demonstrating that virtual and digital induction tools improve accessibility and confidence when clinically needed [13]. The combination of consistent quantitative improvement and supportive qualitative feedback strengthens the credibility of our findings.

Secondary outcomes also showed significant improvement. The secondary outcome measures were informed by existing literature highlighting gaps in junior doctors' understanding of on‑call responsibilities, acute medical workflows, handover processes, and hospital orientation during early clinical transitions [2,5,6,13]. Lack of clarity about the expectations for doctors in their role is an important driver of stress [11]; however, we argue that given each respondent will be a member of the cardiac arrest team, at least on some on-call shifts, full expectation of the role and responsibility is vital. 

Our initial intervention was a hospital tour. It has been found that tours play an important role in improving hospital-based orientation and increasing preparedness for new healthcare professionals [16]. However, with only one attendee, outcome data for PDSA cycle 2 is of limited utility. This adds to the knowledge that while hospital orientation tours have been found to improve preparedness, their effectiveness is highly dependent on timing and relevance [14,15]. This tour was delivered to doctors who had previously worked within the trust, making it less relevant and highlighting the importance of multi‑component induction programmes [5]. PDSA cycle 2 ran in December and reflects recognised issues for doctors rotating outside traditional August and February changeover periods. The GMC has highlighted that these doctors often receive a generic induction that may not meet their needs [13]. Our findings support calls for more tailored induction approaches to improve relevance and avoid inefficient use of resources.

Although our subsequent intervention in PDSA cycle 2 improved preparedness, persistent issues with hospital orientation remained. This suggests that multiple induction strategies are required to address the various contributors to unpreparedness, consistent with systems‑based approaches described in previous literature [5,11]. Delivering in‑person interventions such as tours requires sustained engagement with key stakeholders, as these interventions are resource‑intensive and require coordination, volunteers, and protected time, reinforcing the need for a balanced and sustainable induction model [7].

Limitations

Self‑reported preparedness was chosen as our primary outcome measure, and while this is consistent with most evaluations of induction programmes, the project lacks objective clinical outcomes [5,10]. Unfortunately, self-reported outcomes are susceptible to a number of biases. Firstly, selection bias exists as participation was voluntary, introducing the possibility that respondents differed from non‑respondents (e.g. more engaged or more motivated individuals). To attempt to mitigate this, questionnaires were distributed through multiple channels (email, QR codes, paper at handover) to maximise reach and inclusivity; however, response bias cannot be fully excluded. What's more, recall bias was introduced as questionnaires were completed at least one month after starting on-call duties. This may affect the accuracy of recall regarding initial preparedness; however, this timing was felt to be required to allow sufficient exposure to on‑call duties.

Additionally, although anonymity was ensured with questionnaires, resident doctors were aware of the project and the efforts made by peers to improve induction. Therefore, they may have responded more positively, a limitation well recognised in educational and quality improvement [17]. It is proposed that dissemination of questionnaires by key stakeholders, such as postgraduate medical education, during formal induction may help to mitigate some of these biases in the future.

The use of different cohorts of resident doctors across PDSA cycles limits comparability between groups and increases the risk that observed differences reflect underlying differences in respondents rather than the intervention. Preparedness is subjective and influenced by a number of confounding factors beyond induction. Trainees with more experience of rotational training may feel prepared with limited induction, while others may feel unprepared despite prolonged time within a trust. This variability is well described in the literature on readiness for practice and supports concerns about comparing preparedness across rotating groups composed of individuals with differing experience and confidence thresholds [9,11].

Furthermore, the fact that this study is from a single centre, with small sample sizes, means that the results cannot be generalised to a larger population of resident doctors. Also, the lack of a validated measurement tool and the local development of a questionnaire raise the possibility of measurement error, which could have contributed to baseline data missing a single response for the secondary outcome measurement of hospital orientation.

## Conclusions

This quality improvement project adds to existing evidence that structured induction improves doctors' perceived preparedness and confidence for on-call duties. The introduction of a written induction booklet was associated with meaningful improvements in resident doctors' preparedness for general medical on-call shifts at Salford Royal Hospital. However, our findings suggest that a variety of structured induction materials are required to ensure adequate preparation and should be tailored to the needs of the doctors rotating. Furthermore, ensuring induction materials within a timely manner is crucial and an area for further improvement.

The impact of preparedness on clinician performance has been discussed, but there is scope for further research on the impact of preparedness on clinical outcomes. 

## Appendices

Appendix A

**Table 3:** Questionnaire used for baseline data collection and PDSA cycle 2 Medical Staff Induction to On‑Call Shifts at Salford Royal Hospital questionnaire used in the initial data collection and data collection in cycle 2 (post-intervention). This was distributed online and also printed. PDSA: Plan-Do-Study-Act; RMO: receiving medical officer

Question	Response type
When did you start at Salford Royal Hospital?	Free‑text
What grade are you?	Free‑text

Question	Response options
Did you receive a trust/hospital induction when you started at Salford Royal Hospital?	Yes/No
Did you receive an induction for general medicine on‑call duties when you started at Salford Royal Hospital?	Yes/No

Statement	1	2	3	4	5
I understood the different on‑call shifts at Salford Royal and my role	☐	☐	☐	☐	☐
I understood how the acute medical take worked at Salford Royal	☐	☐	☐	☐	☐
I knew where and when handovers were and whether I was expected to attend	☐	☐	☐	☐	☐
I knew how to access senior support out of hours	☐	☐	☐	☐	☐
The information provided was easy to understand and up to date	☐	☐	☐	☐	☐
I felt I had enough information to safely start on‑call duties/felt prepared	☐	☐	☐	☐	☐

Question	Response options
In your first month of on‑calls, how often did you get lost or ask for directions?	Every shift/Every other shift/Rarely/Never
During your time at Salford, how often have you heard of a patient being admitted under the wrong specialty?	Daily/Weekly/Rarely/Never

Question	Response options
Do you think general medical induction needs improvement or updating?	Yes/No

Proposed induction content	Selected
Different on‑call shifts and the team on each	☐
How bleeps work at Salford	☐
Cardiac arrest information (coverage and alerting)	☐
The acute medical take, including what should be accepted as RMO	☐
Handover locations and times	☐
Different wards and specialties within the hospital	☐

Question
Do you have any other comments on induction at Salford Royal or anything else that would have been useful?
Do you have any other comments on the induction tour you attended?

Appendix B

**Table 4:** Questionnaire used for PDSA cycle 1 (hospital orientation tour) Medical Staff Induction to On‑Call Shifts at Salford Royal Hospital questionnaire sent to attendees of the tour in cycle 1 (following the first intervention). PDSA: Plan-Do-Study-Act

Question	Response type
When did you start at Salford Royal Hospital?	Free‑text
What grade are you?	Free‑text

Question	Response options
Did you find the hospital tour useful in preparing for on‑call duties?	Yes/No/Not sure
Do you think the tour should be offered to all new starters?	Yes/No/Not sure

Question	Response options
In your first month of on‑calls, how often did you get lost or ask for directions?	Every shift/Every other shift/Rarely/Never

Question
Do you have any other comments on the induction tour you attended?
